# Evaluation of the detection ability of uropathogen morphology and vaginal contamination by the Atellica UAS800 automated urine microscopy analyzer and its effectiveness

**DOI:** 10.1002/jcla.23698

**Published:** 2021-01-10

**Authors:** Akihiro Nakamura, Tetsuya Shinke, Nobuyoshi Noguchi, Masaru Komatsu, Hachiro Yamanishi

**Affiliations:** ^1^ Department of Clinical Laboratory Science Faculty of Health Care Tenri Health Care University Tenri Japan; ^2^ Department of Clinical Bacteriology Clinical Laboratory Medicine Tenri Hospital Tenri Japan

**Keywords:** automated urine microscopy analysis, multivariable logistic regression analysis, urinary tract infection, uropathogens morphology, vaginal contamination

## Abstract

**Background:**

To help combat the worldwide spread of multidrug‐resistant Enterobacterales, which are responsible for many causes of urinary tract infection (UTI), we evaluated the ability of the Atellica UAS800 automated microscopy system, the only one offering the capability of bacterial morphological differentiation, to determine its effectiveness.

**Methods:**

We examined 118 outpatient spot urine samples in which pyuria and bacteriuria were observed using flow cytometry (training set: 81; cross‐validation set: 37). The ability of the Atellica UAS800 to differentiate between bacilli and cocci was verified. To improve its ability, multiple logistic regression analysis was used to construct a prediction formula.

**Results:**

This instrument's detection sensitivity was 106 CFU/ml, and reproducibility in that range was good, but data reliability for the number of cocci was low. Multiple logistic regression analysis with each explanatory variable (14 items from the Atellica UAS800, age and sex) showed the best prediction formula for discrimination of uropathogen morphology was a model with 5 explanatory variables: number of bacilli (*p* < 0.001), squamous epithelial cells (*p* = 0.004), age (*p* = 0.039), number of cocci (*p* = 0.107), and erythrocytes (*p* = 0.111). For a predicted cutoff value of 0.449, sensitivity was 0.879 and specificity was 0.854. In the cross‐validation set, sensitivity was 0.813 and specificity was 0.857.

**Conclusions:**

The Atellica UAS800 could detect squamous epithelial cells, an indicator of vaginal contamination, with high sensitivity, which further improved performance. Simultaneous use of this probability prediction formula with urinalysis results may facilitate real‐time prediction of uropathogens and vaginal contamination, thus providing helpful information for empiric therapy.

## Graphical abstract

The workflow and its performance for discrimination of bacterial morphology and vaginal contamination using Atellica UAS800 are shown. The ability to discriminate bacterial morphology (positive predictive value) was 91% in the bacilli group and 81% in the cocci or polymicrobial group in the training set. The ability to discriminate vaginal contamination (positive predictive value) was 96% in the non‐contamination group and 84% in the contamination group within the training set. In the cross‐validation set, the positive predictive value was 82% in the bacilli group, 80% in the cocci or polymicrobial group, 92% in the vaginal non‐contamination group, and 80% in the contamination group.

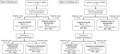

## 1 INTRODUCTION

Urinary tract infection (UTI) is an infectious disease frequently encountered in daily life and is a representative infection that can cause serious sepsis.^1^ The multidrug‐resistant Enterobacteriaceae, which produce extended‐spectrum β‐lactamase (ESBL) and carbapenemase, have recently spread worldwide.^2^, ^3^, ^4^ The risk factor of intestinal colonization of multidrug‐resistant Enterobacteriaceae is the use of broad‐spectrum antibacterial drugs such as fluoroquinolone and carbapenem.^5^, ^6^, ^7^ In addition, UTIs are very difficult to diagnose, and unnecessary treatment with antibacterial drugs of conditions with positive urine culture due to asymptomatic bacteria and vulvar‐contaminated urine is often wasteful.^8^

Currently, the gold standard method of identifying the causative organism of UTI is by bacterial culture, but such tests require long testing time, and obtaining test results concurrently with outpatient treatment is generally impossible. Therefore, in most UTIs, antimicrobial therapy is presently performed without knowing the causative organism. In addition, Gram staining, which is a rapid test, is complicated, and its specimen processing capacity is poor. A system in which the causative organism of UTI can be predicted from automated urinary analysis, which is the initial tool used in the diagnosis of UTI, is needed.

In recent years, automated microscopy has become the main tool currently used worldwide for automated urinary analysis.^9^ The Atellica UAS800 (Siemens K.K., Tokyo, Japan), which was evaluated in this study, is an automated urine microscopy analyzer whose method is based on the principle of capturing and analyzing microscopic images with a digital camera. The Atellica UAS800 can measure 14 items: total bacteria (BAC), bacilli (BACr), cocci (BACc), erythrocytes (RBC), leukocytes (WBC), hyaline casts (HYA), squamous epithelial cells (EPI), nonsquamous epithelial cells (NEC), crystals (CRY), leukocyte clumps (WBCc), pathological casts (PAT), yeasts (YEA), mucus (MUC), and sperm (SPRM). In addition, it is the only morphological instrument that distinguishes urinary tract pathogens into bacilli and cocci, but its performance has not been evaluated.

Therefore, the purpose of this study was to evaluate the bacterial detection and bacterial morphological discrimination ability of the Atellica UAS800 automated microscopy analysis system and to calculate a prediction equation using multiple logistic regression analysis to improve its ability.

## 2 MATERIALS AND METHODS

### 2.1 Materials

Among fresh outpatient urine samples submitted to the general urinalysis laboratory of Tenri Hospital (a 1001‐bed primary care hospital in Nara, Japan) between March and April in 2018, 118 samples (from 47 men and 71 women) were chosen in which pyuria (>5–10 WBCs/high‐power field) was confirmed and bacteriuria (>1+) was observed based on microscopic testing. Among them, 37 samples (from 14 men and 23 women) were defined as the cross‐validation set. This study was approved by the ethical committees of Tenri Hospital and Tenri Health Care University (approval nos. 899 and 115, respectively).

### 2.2 Measurement of automated microscopy

We used the Atellica UAS800 system to qualitatively measure the following 14 items in the target samples: number of BAC, BACr, BACc, YEA, RBC, WBC, WBCc, NEC, EPI, PAT, HYA, MUC, SPRM, and CRY.

### 2.3 Verification of detection sensitivity and reproducibility using ATCC strains

To test the detection sensitivity and reproducibility of the Atellica UAS800, we used *Escherichia coli* ATCC25922 and *Staphylococcus aureus* ATCC25923. For the detection sensitivity test, bacterial dilutions of 10^0^ colony‐forming units (CFU)/ml to 108 CFU/ml were prepared, and the linearity of the mean of three measurements was confirmed. The reproducibility test was performed five times using bacterial solutions at concentrations above the detection sensitivity, and the coefficient of variation was calculated and evaluated.

### 2.4 Microbiologic testing

We performed Gram‐stain microscopy analysis and urinary culture. For urinary culture, we inoculated 5% sheep blood agar/Drigalski medium with 10 μl of fresh urine using a loop and aerobically cultured each sample at 37°C for 18 to 24 h. We conducted strain identification of the grown colonies by matrix‐assisted laser desorption ionization time‐of‐flight mass spectrometry (MALDI‐TOF MS). We used a MALDI Biotyper (Bruker Daltonik, Bremen, Germany) and conducted ethanol‐formic acid protein extraction as the pretreatment method. The quantification of bacteria by Gram staining and urine culture was as previously reported.^10^

### 2.5 Statistical analysis

To distinguish between the bacilli group, cocci group, and polymicrobial group, we performed bivariate analysis on the basis of the 14 qualitative urinalysis items and age and sex as explanatory variables and each bacterial morphology by Gram staining as the response variable using training set data. We further investigated the discriminant characteristics of three items, BAC, BACr, and BACc, with receiver operating characteristic curve (ROC) analysis. In addition, we performed multiple logistic regression analysis using the 14 qualitative urinalysis items and age and sex. Multiple logistic regression was also performed to construct predictive equations to improve the ability to differentiate bacterial morphology. Moreover, the calculated prediction formula using multiple logistic regression analysis was verified in the cross‐validation set. In addition, we used the stepwise method to select explanatory variables in the multiple logistic regression analysis.

To distinguish between the vaginal contamination group and non‐contamination group, as described above, we performed bivariate analysis, multiple logistic regression analysis, and ROC analysis on the basis of the results of vaginal contamination as judged using Gram staining and culture analysis.

We used StatFlex Ver. 6.0 (Artech Co., Ltd., Osaka, Japan) software for the statistical analysis, and the level of significance was set at *p* = 0.05.

## 3 RESULTS

### 3.1 Verification of detection sensitivity and reproducibility using ATCC strains

**FIGURE 1:**
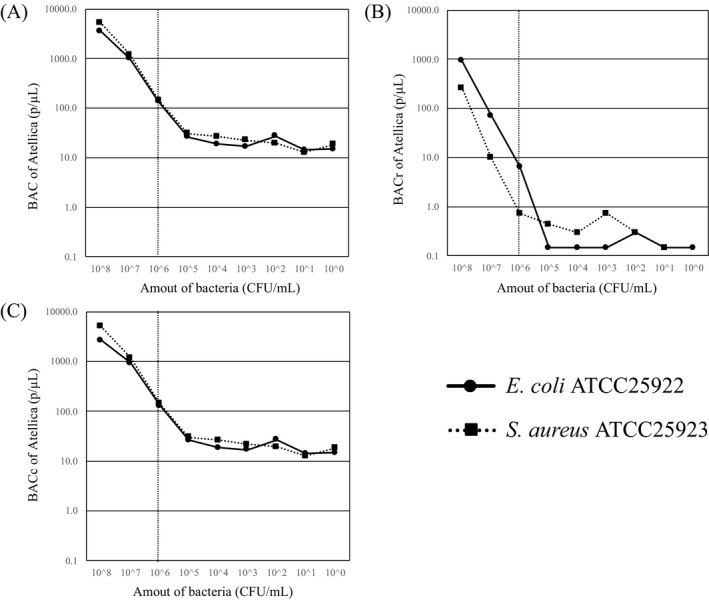
Results of verification of detection sensitivity of the Atellica UAS800 using ATCC strains. (A) BAC, (B) BACr, and (C) BACc

Results of verification of the detection sensitivity of the Atellica UAS800 using the ATCC strains are shown in Figure 1. The detection limit of the Atellica UAS800 was 106 CFU/ml for both bacilli and cocci. Results of reproducibility for bacteria counts above 106 CFU/ml are shown in Table 1. Reproducibility in that range was good, with an average of coefficient of variation percent (CV%) = 10.3, but the reliability of the BACc value was low because this value, which indicates the number of cocci, was high even when *E. coli* ATCC25923 was measured.

**TABLE 1:** Results of reproducibility of BAC, BACr and BACc values by the Atellica UAS800 using ATCC strains

Item	Strain	Conc. (CFU/ml)	Number of measurements (p/µl)					Average ±SD	CV (%)
			1	2	3	4	5		
BAC	*E. coli* ATCC25922	10^8^	4140.4	4180.0	4294.8	4189.2	4279.9	4216.9 ± 60	1.4
		10^7^	1027.4	1089.9	1160.3	1083.7	1011.1	1074.5 ± 52.8	4.9
		10^6^	143.9	122.3	147.4	131.6	120.6	133.1 ± 10.9	8.2
	*S. aureus* ATCC25923	10^8^	5658.0	5072.8	5841.0	5333.2	5129.5	5406.9 ± 298.5	5.5
		10^7^	1070.1	1132.6	1046.8	1031.8	1012.0	1058.6 ± 41.5	3.9
		10^6^	116.2	120.6	127.2	114.8	118.8	119.5 ± 4.3	3.6
BACr	*E. coli* ATCC25922	10^8^	783.6	671.0	662.2	623.0	700.5	688.1 ± 53.8	7.8
		10^7^	47.5	47.1	47.5	40.9	41.4	44.9 ± 3.1	6.8
		10^6^	4.4	4.4	4.8	5.7	6.2	5.1 ± 0.7	14.0
	*S. aureus* ATCC25923	10^8^	278.5	243.3	236.7	221.3	213.0	238.6 ± 22.7	9.5
		10^7^	11.4	9.2	9.2	9.2	9.2	9.7 ± 0.9	9.1
		10^6^	0.4	0.0	1.3	1.8	0.4	0.8 ± 0.6	81.6
BACc	*E. coli* ATCC25922	10^8^	3356.8	3509.0	3632.6	3566.2	3579.4	3528.8 ± 94.6	2.7
		10^7^	979.9	1042.8	1112.8	1042.8	969.8	1029.6 ± 51.6	5.0
		10^6^	139.5	117.9	142.6	125.8	114.4	128.0 ± 11.3	8.8
	*S. aureus* ATCC25923	10^8^	5379.4	4829.4	5604.3	5111.9	4916.6	5168.3 ± 288.5	5.6
		10^7^	1058.6	1123.3	1037.5	1022.6	1002.8	1049.0 ± 41.4	4.0
		10^6^	115.7	120.6	125.8	113.1	118.4	118.7 ± 4.4	3.7

Abbreviations: BAC, number of total bacteria; BACc, number of cocci; BACr, number of bacilli; CFU, colony‐forming unit; CV, coefficient of variation (CV% = standard deviation/mean×100).

### 3.2 Microbiologic test results in the target specimens

Following Gram staining of the 81 target specimens in the training set, only bacilli were detected in 48 samples (Gram‐negative rods [GNR]: 37 samples, Gram‐positive rods [GPR]: 7 samples, GNR +GPR: 4 samples), only cocci were detected in 22 samples (all Gram‐positive cocci [GPC]), and both bacilli and cocci were detected in 11 samples. In the cultures, only a single species of bacteria was detected in 61 specimens (single‐species group), whereas 2 or more strains were detected in 20 specimens (polymicrobial group). In the single‐species group, bacilli were detected in 34 specimens (bacilli group), which included the following bacterial strains: *E. coli*, 24 strains; *Klebsiella pneumoniae*, 3 strains; *Enterobacter cloacae* and *Serratia marcescens*, 2 strains; and *Acinetobacter baumannii*, *Citrobacter koseri*, and *Proteus rettgeri*, 1 strain each. In contrast, cocci were detected in 27 specimens (cocci group), which included coagulase‐negative *Staphylococcus* including *S. epidermidis*, *S. caprae*, and *S. haemolyticus*, 11 strains; *Enterococcus faecalis*, 6 strains; *S*. *aureus*, 5 strains; *Streptococcus agalactiae* and *Streptococcus viridans* group, 2 strains; and *Candida krusei*, 1 strain.

Among the 37 target specimens in the cross‐validation set, following Gram staining, only bacilli were detected in 21 samples (GNR: 16 samples, GNR +GPR: 5 samples), only cocci were detected in 10 samples (all GPC), and both bacilli and cocci were detected in 6 samples. In the cultures, only a single species of bacteria was detected in 28 specimens (single‐species group), whereas 2 or more strains were detected in 9 specimens (polymicrobial group). In the single‐species group, bacilli were detected in 17 specimens (bacilli group), which included the following bacterial strains: *E. coli*, 10 strains; *K. pneumoniae*, 3 strains; and *K. oxytoca*, *C. koseri*, *Enterobacter aerogenes*, and *Corynebacterium striatum*, 1 strain each. In contrast, cocci were detected in 11 specimens (cocci group), which included *E. faecalis*, 5 strains; *S. epidermidis*, 2 strains; and *Aerococcus urinae*, *Enterococcus faecium*, *S. agalactiae*, and *S. aureus*, 1 strain each.

### 3.3 Results of vaginal contamination following Gram staining and urinary culture

Among the 81 target specimens in the training set, 60 specimens were included in the non‐contamination group, and 21 were included in the contamination group. Among the 37 target specimens in the cross‐validation set, 28 specimens were included in the non‐contamination group, and 9 were included the contamination group.

### 3.4 Bivariate and ROC analysis using the bacilli group, cocci group, and polymicrobial group in training set data

**FIGURE 2:**
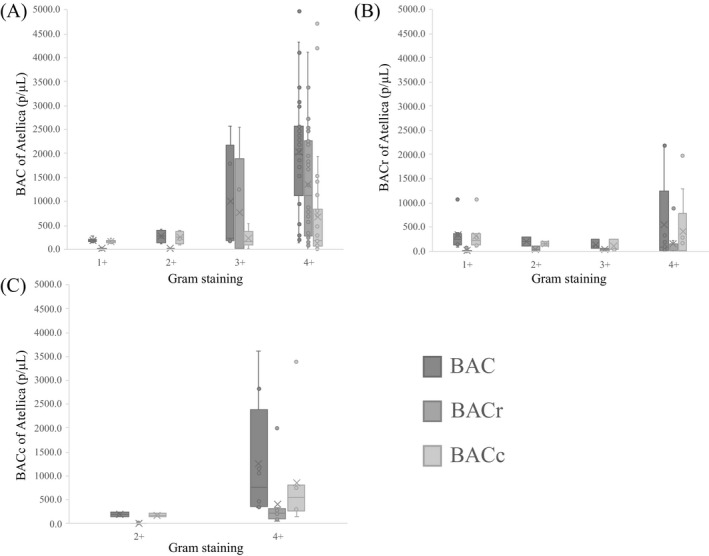
Box‐and‐whisker plots of Gram staining and BAC, BACr and BACc values by the Atellica UAS800. (A) Bacilli group, *n* = 48, (B) cocci group, *n* = 22, and (C) polymicrobial group, *n* = 11

**FIGURE 3:**
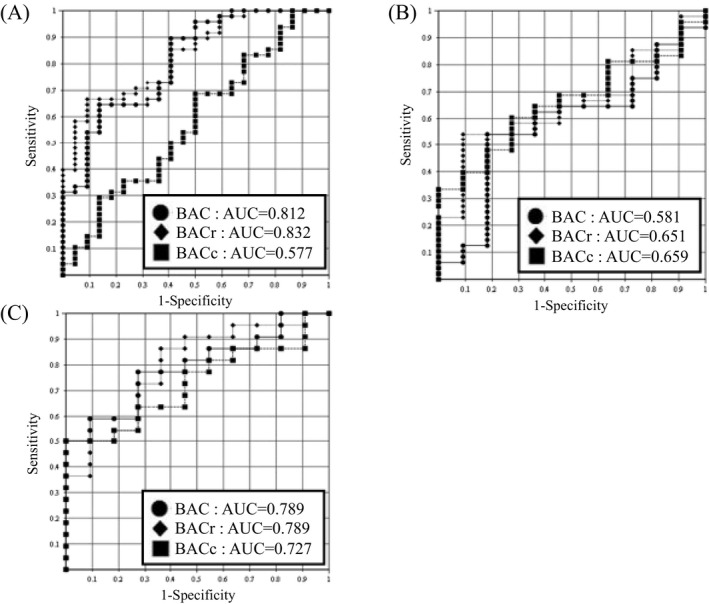
ROC analysis of BAC, BACr and BACc values by the Atellica UAS800. (A) Bacilli vs. cocci, (B) bacilli vs. polymicrobial, and (C) cocci vs. polymicrobial

The results of bivariate analysis using the bacilli group, cocci group, and polymicrobial group in training set data are shown in Table 2, and the box‐and‐whisker plots of Gram staining and the BAC, BACr, and BACc values from the Atellica UAS800 are shown in Figure 2. The items with significant differences were sex (bacilli vs. cocci, *p* = 0.002; bacilli vs. polymicrobial, *p* = 0.013), BAC (bacilli vs. cocci, *p* < 0.001), BACr (bacilli vs. cocci, *p* < 0.001), YEA (bacilli vs. cocci, *p* = 0.002), RBC (bacilli vs. cocci, *p* = 0.050; cocci vs. polymicrobial, *p* = 0.032), and EPI (bacilli vs. cocci, *p* = 0.002). The cocci group and polymicrobial group did not differ in characteristics, and it was difficult to distinguish between these two groups. The results of their ROC analyses are shown in Figure 3. BAC and BACr showed moderate discrimination ability (BAC, area under the curve [AUC] =0.812; BACr, AUC =0.832) in distinguishing between bacilli and cocci, whereas the others did not. Bivariate analysis did not provide the capability for high‐performance differentiation.

**TABLE 2:** Bivariate analysis of the distinguishability of the bacilli group, cocci group and polymicrobial group using the Atellica UAS800

Item	Mean ±SD or *n* (%)			*p* value*		
	Bacilli group (*n* = 48)	Cocci group (*n* = 22)	Polymicrobial group (*n* = 11)	Bacilli vs. cocci	Bacilli vs. polymicrobial	Cocci vs. polymicrobial
Age (years)	69.1 ± 14.6	74.4 ± 10.3	76.3 ± 10.6		0.144	0.540
Sex (male)	12 (25.0)	14 (63.6)	7 (63.6)	**0.002**	**0.013**	1.000
Atellica data (p/µl)						
BAC	1513.3 ± 1278.6	389.7 ± 623.5	930.9 ± 1195.9	***<0.001***	0.235	0.052
BACc	519.7 ± 937.6	316.5 ± 494.0	635.1 ± 956.1	0.306	0.321	0.152
BACr	993.6 ± 1115.7	73.3 ± 188.9	295.8 ± 580.5	***<0.001***	0.059	0.054
YEA	0.7 ± 2.0	6.7 ± 16.1	23.5 ± 67.6	**0.002**	0.263	0.405
RBC	10.0 ± 21.8	56.4 ± 195.8	4.6 ± 9.0	**0.050**	0.199	**0.032**
WBC	248.5 ± 381.1	693.2 ± 981.0	178.2 ± 121.5	0.869	0.330	0.620
WBCc	6.2 ± 14.4	49.4 ± 99.5	47.8 ± 125.0	0.847	0.173	0.485
NEC	1.0 ± 1.5	0.7 ± 1.2	0.9 ± 1.7	0.207	0.383	0.887
EPI	14.8 ± 21.2	3.7 ± 7.5	3.7 ± 4.5	**0.002**	0.151	0.244
PAT	1.1 ± 4.0	1.1 ± 1.8	7.5 ± 15.2	0.065	0.185	0.703
HYA	0.5 ± 1.0	0.5 ± 0.9	0.3 ± 0.3	0.600	0.536	0.966
MUC	82.2 ± 133.6	104.4 ± 130.5	29.2 ± 51.6	0.455	0.081	0.095
SPRM	0.0 ± 0.0	0.0 ± 0.0	0.0 ± 0.0	‐	‐	‐
CRY	1.6 ± 4.6	0.6 ± 1.3	11.5 ± 33.5	0.566	0.245	0.137

Abbreviations: BAC, number of total bacteria; BACc, number of cocci; BACr, number of bacilli; CRY, crystals; EPI, squamous epithelial cells; HYA, hyaline cast; MUC, mucus; NEC, nonsquamous epithelial cells; PAT, pathological cast; RBC, erythrocytes; SPRM, sperm; WBC, leukocytes; WBCc, leukocyte clumps; YEA, yeasts.

* Bold Italic numbers indicate *p* < 0.001; bold numbers indicate *p* < 0.05.

### 3.5 Multiple logistic regression analysis for discrimination of the bacilli group and cocci or polymicrobial group

The results of final model selection using multiple logistic regression analysis for discrimination of the bacilli group and cocci or polymicrobial group are shown in Table 3. The best model was model 1, which included the 5 items of BACr, EPI, age, BACc, and RBC (Akaike information criterion [AIC] =72.3, AUC =0.913) and showed high discrimination ability. Therefore, we choose this as the model for calculation of the probability prediction formula to differentiate between the bacilli group and cocci or polymicrobial group. The probability prediction formula was calculated as follows:

$$\text{Prediction}\;\text{value}\;Y=1/\{1+e^{‐(2.7025‐0.0025\times \text{BACr}‐0.1072\times \text{EPI}+0.06061\times \text{age}‐0.0005\times \text{BACc + 0}.00562\times \text{RBC})}\}$$

**TABLE 3:** Results of final model selection using multiple logistic regression analysis for discrimination of bacilli group and cocci or polymicrobial group

Models	Variables	β	SE (β)	*p* value	OR (95% CI)	AIC	AUC
Model 1 (final model)	α (constant)	−2.7025	2.12865	‐	‐	72.3	0.913
	BACr	−0.0025	0.00076	***<0.001***	0.99750 (0.99602–0.99898)		
	EPI	−0.1072	0.03716	**0.004**	0.89837 (0.83527–0.96624)		
	Age	0.06061	0.02931	**0.039**	1.06248 (1.00318–1.12530)		
	BACc	−0.0005	0.00034	0.107	0.99946 (0.99880–1.00012)		
	RBC	0.00562	0.00353	0.111	1.00564 (0.99870–1.01262)		
Model 2	α (constant)	−2.8542	2.09789	‐	‐	73.3	0.903
	BACr	−0.0026	0.00083	**0.002**	0.99744 (0.99582–0.99906)		
	EPI	−0.095	0.03467	**0.006**	0.90940 (0.84966–0.97334)		
	Age	0.05675	0.02844	**0.046**	1.05839 (1.00101–1.11906)		
	RBC	0.00565	0.00364	0.120	1.00566 (0.99852–1.01286)		
Model 3	α (constant)	−2.0209	2.31733	‐	‐	74.7	0.904
	BACr	−0.0025	0.00082	**0.002**	0.99751 (0.99591–0.99912)		
	EPI	−0.0816	0.03665	**0.026**	0.92165 (0.85778–0.99029)		
	Age	0.04867	0.02989	0.104	1.04988 (0.99013–1.11323)		
	RBC	0.00541	0.00367	0.141	1.00543 (0.99822–1.01269)		
	Sex	−0.6146	0.73887	0.406	0.54086 (0.12710–2.30152)		
Model 4	α (constant)	−1.5543	1.94337	‐	‐	74.6	0.896
	BACr	−0.0025	0.00074	***<0.001***	0.99753 (0.99608–0.99898)		
	EPI	−0.0903	0.03124	**0.004**	0.91363 (0.85936–0.97132)		
	Age	0.04504	0.02653	0.090	1.04607 (0.99307–1.10190)		
	BACc	−0.0005	0.00033	0.105	0.99946 (0.99881–1.00011)		
Model 5	α (constant)	−1.5543	1.94337	‐	‐	74.6	0.896
	BACr	−0.0019	0.00082	**0.019**	0.99807 (0.99646–0.99968)		
	EPI	−0.0903	0.03124	**0.004**	0.91363 (0.85936–0.97132)		
	Age	0.04504	0.02653	0.090	1.04607 (0.99307–1.10190)		
	BAC	−0.0005	0.00033	0.105	0.99946 (0.99881–1.00011)		

Abbreviations: AIC, Akaike information criterion; AUC, area under the curve; BAC, number of total bacteria; BACc, number of cocci; BACr, number of bacilli; CI, confidence interval; EPI, squamous epithelial cells; OR, odds ratio; RBC, erythrocytes; SE, standard error.

* Bold Italic numbers indicate *p* < 0.001; bold numbers indicate *p* < 0.05.

When the cutoff value for the predicted prediction value Y was 0.449, the sensitivity was 0.879 and the specificity was 0.854 (Table 4). In addition, in the cross‐validation set, the sensitivity was 0.813 and the specificity was 0.857.

**TABLE 4:** Sensitivity and specificity in discrimination of bacilli group and cocci or polymicrobial group

Prediction value	Training set		Validation set	
	Cocci or polymicrobial (*n* = 33)	Bacilli (*n* = 48)	Cocci or polymicrobial (*n* = 16)	Bacilli (*n* = 21)
Y > 0.449	29	7	13	3
Y ≤ 0.449	4	41	3	18
Sensitivity	0.879		0.813	
Specificity	0.854		0.857	

### 3.6 Statistical analysis of the distinguishability of vaginal contamination

Results of the bivariate analysis of the distinguishability of vaginal contamination using the Atellica UAS800 are shown in Table 5. The item with the highest discrimination function was EPI (AUC =0.878, *p* < 0.001), followed by NEC (AUC =0.680, *p* = 0.012), with EPI showing moderate discrimination ability. The results of final model selection using multiple logistic regression analysis for the discrimination of vaginal contamination are shown in Table 6. The best model was model 1, which included the 4 items of EPI, MUC, PAT, and WBCc (AIC =52.5, AUC =0.933) and showed high discrimination ability. Therefore, we chose this as the model for calculation of the probability prediction formula to differentiate between the contamination group and non‐contamination group. The probability prediction formula was calculated as follows:

$$\text{Prediction}\;\text{value}\;Y=1/\{1+e^{‐(2.1454+0.2029\times \text{EPI}‐0.0088\times \text{MUC}+0.12472\times \text{PAT}‐0.1644\times \text{WBCc})}\}$$

**TABLE 5:** Bivariate analysis of the distinguishability of vaginal contamination using automated urine microscopy analysis

Item	Mean ±SD or *n* (%)		*p* value	AUC	Cutoff value	Sensitivity
	Contamination (*n* = 21)	Non‐contamination (*n* = 60)				
Age (years)	66.2 ± 17.9	73.4 ± 10.8	0.300	0.576	75.2	0.524
Sex (male)	1 (4.8)	31 (51.7)	***<0.001***	‐	‐	0.905
Atellica data (p/µl)						
BAC	919.5 ± 1207.9	1202.4 ± 1224.2	0.594	0.539	319.5	0.617
BACc	380.0 ± 706.1	515.2 ± 885.4	0.601	0.538	194.2	0.433
BACr	539.4 ± 1049.2	687.1 ± 962.3	0.553	0.544	68.9	0.619
YEA	1.7 ± 6.5	6.7 ± 30.5	0.420	0.550	0.3	0.530
RBC	12.8 ± 26.9	25.0 ± 119.9	0.711	0.527	3.0	0.520
WBC	118.6 ± 116.7	444.1 ± 697.0	0.164	0.602	100.4	0.550
WBCc	2.2 ± 5.1	31.1 ± 82.0	**0.023**	0.665	1.2	0.612
NEC	1.5 ± 1.9	0.8 ± 1.1	**0.012**	0.680	0.4	0.620
EPI	29.7 ± 24.5	3.4 ± 6.2	***<0.001***	0.878	4.7	0.810
PAT	3.5 ± 11.1	1.5 ± 4.1	0.173	0.590	0.0	0.000
HYA	0.8 ± 1.2	0.4 ± 0.8	0.059	0.621	0.0	0.000
MUC	126.4 ± 161.4	65.2 ± 107.8	0.104	0.619	41.8	0.633
SPRM	0.0 ± 0.0	0.0 ± 0.0	‐	‐	‐	‐
CRY	3.5 ± 6.7	2.4 ± 14.5	0.135	0.576	0.0	0.000

Abbreviations: AUC, area under the curve; BAC, number of total bacteria; BACc, number of cocci; BACr, number of bacilli; CRY, crystals; EPI, squamous epithelial cells; HYA, hyaline cast; MUC, mucus; NEC, nonsquamous epithelial cells; PAT, pathological cast; RBC, erythrocytes; SPRM, sperm; WBC, leukocytes; WBCc, leukocyte clumps; YEA, yeasts.

* Bold Italic numbers indicate *p* < 0.001; bold numbers indicate *p* < 0.05.

**TABLE 6:** Results of final model selection for discrimination of vaginal contamination

Models	Variables	β	SE (β)	*p* value	OR (95% CI)	AIC	AUC
Model 1 (final model)	α (constant)	−2.1454	0.59205	‐	‐	52.5	0.933
	EPI	0.2029	0.05558	***<0.001***	1.22495 (1.09852–1.36593)		
	MUC	−0.0088	0.00442	**0.046**	0.99122 (0.98268–0.99984)		
	PAT	0.12472	0.06777	0.066	1.13283 (0.99193–1.29375)		
	WBCc	−0.1644	0.14257	0.249	0.84840 (0.64157–1.12190)		
Model 2	α (constant)	−3.0318	1.14288	‐	‐	53.1	0.939
	EPI	0.17384	0.05694	**0.002**	1.18987 (1.06421–1.33036)		
	PAT	0.1415	0.07635	0.064	1.15200 (0.99189–1.33795)		
	MUC	−0.0079	0.00426	0.064	0.99213 (0.98388–1.00045)		
	WBCc	−0.1998	0.15981	0.211	0.81887 (0.59866–1.12007)		
	Sex	1.40001	1.31535	0.287	4.05524 (0.30788–53.4131)		
Model 3	α (constant)	−2.1289	0.59897	‐	‐	54.5	0.933
	EPI	0.20597	0.05933	***<0.001***	1.22871 (1.09383–1.38023)		
	MUC	−0.0088	0.00444	**0.047**	0.99123 (0.98264–0.99989)		
	PAT	0.12305	0.06819	0.071	1.13094 (0.98946–1.29264)		
	WBCc	−0.1611	0.14275	0.259	0.85118 (0.64344–1.12600)		
	NEC	−0.0503	0.30866	0.871	0.95099 (0.51933–1.74144)		
Model 4	α (constant)	−2.7609	0.54933	‐	‐	55.7	0.906
	EPI	0.19188	0.05015	***<0.001***	1.21152 (1.09809–1.33666)		
	MUC	−0.0063	0.00384	0.100	0.99370 (0.98624–1.00121)		
	PAT	0.06703	0.04139	0.105	1.06933 (0.98600–1.15971)		
Model 5	α (constant)	−3.2005	1.03766	‐	‐	59.7	0.911
	EPI	0.11582	0.03851	**0.003**	1.12280 (1.04117–1.21082)		
	Sex	1.5137	1.15298	0.189	4.54352 (0.47421–43.5324)		
	WBCc	−0.023	0.03655	0.530	0.97728 (0.90971–1.04986)		
	NEC	−0.1496	0.24822	0.547	0.86102 (0.52934–1.40054)		

Abbreviations: AIC, Akaike information criterion; AUC, area under the curve; CI, confidence interval; EPI, squamous epithelial cells; MUC, mucus; NEC, nonsquamous epithelial cells; OR, odds ratio; PAT, pathological cast; SE, standard error; WBCc, leukocyte clumps.

* Bold Italic numbers indicate *p* < 0.001; bold numbers indicate *p* < 0.05.

When the cutoff value for the predicted prediction value Y was 0.159, the sensitivity was 0.905 and the specificity was 0.900 (Table 7), and in the cross‐validation set, the sensitivity was 0.889 and the specificity was 0.857.

**TABLE 7:** Sensitivity and specificity in the discrimination of vaginal contamination

Prediction value	Training set		Validation set	
	Contamination (*n* = 21)	Non‐contamination (*n* = 60)	Contamination (*n* = 9)	Non‐contamination (*n* = 28)
Y > 0.159	19	6	8	4
Y ≤ 0.159	2	54	1	24
Sensitivity	0.905		0.889	
Specificity	0.900		0.857	

### 3.7 Workflow for discrimination of bacterial morphology and vaginal contamination

**FIGURE 4:**
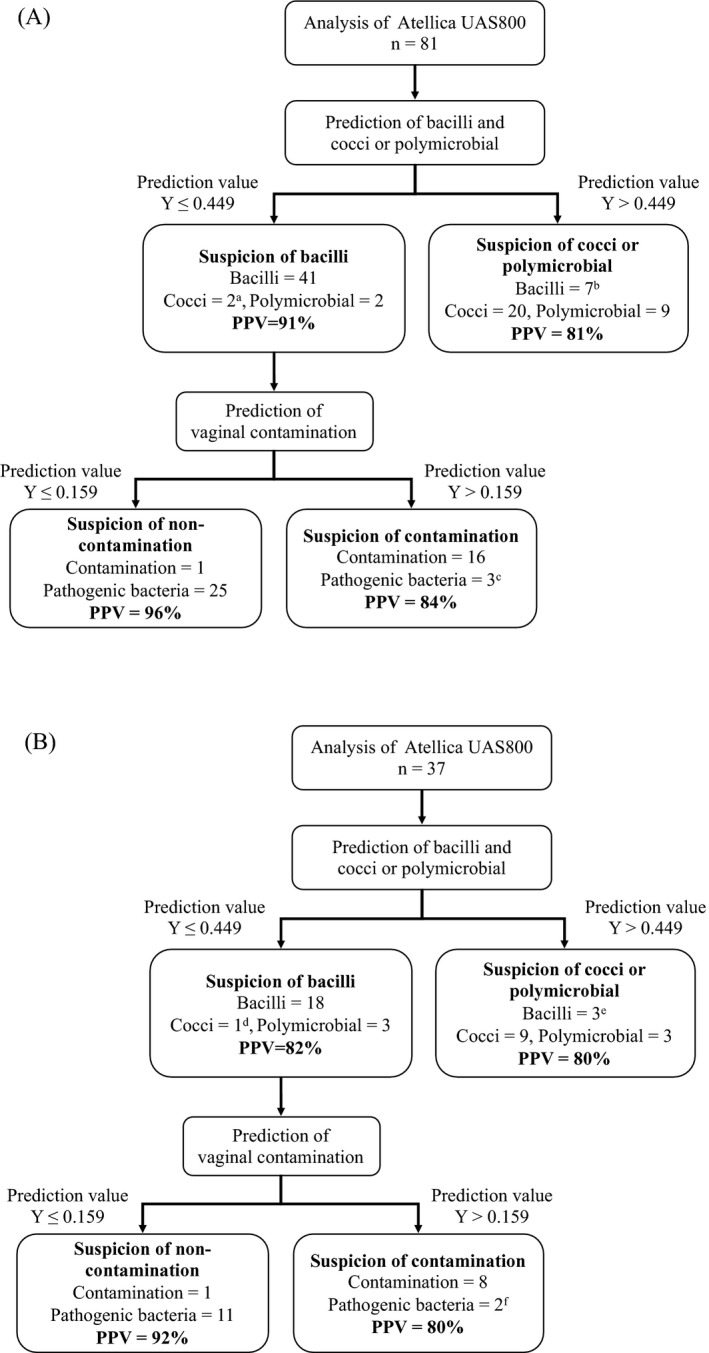
Work flow for discrimination of bacterial morphology and vaginal contamination. (A) Data of the training set (*n* = 81). (B) Data of the validation set (*n* = 37). ^a^ *Enterococcus faecalis*, *Streptococcus agalactiae* (1 each), ^b^ *Escherichia coli* (3), Kleb/Entero group (2), others (2), ^c^ *E. coli* (2), *Candida krusei* (1), ^d^ *E. faecalis* (1), ^e^ *E. coli* (2), *Citrobacter koseri* (1), ^f^ Kleb/Entero group (2). PPV, positive predictive value

The workflow and its performance for discrimination of bacterial morphology and vaginal contamination are shown in Figure 4. In the training set, the ability to discriminate bacterial morphology (positive predictive value [PPV]) was 91% in the bacilli group and 81% in the cocci or polymicrobial group, whereas the ability to discriminate vaginal contamination (PPV) was 96% in the non‐contamination group and 84% in the contamination group. In the cross‐validation set, the PPV was 82% in the bacilli group, 80% in the cocci or polymicrobial group, 92% in the vaginal non‐contamination group, and 80% in the contamination group.

## 4 DISCUSSION

Recently, drug‐resistant bacteria including ESBL‐ and carbapenemase‐producing Enterobacteriaceae have spread remarkably worldwide. These drug‐resistant bacteria have been detected in urine samples at high rates, and the use of broad‐spectrum antimicrobial agents represented by fluoroquinolone antibacterials is an associated risk factor. In recent years, automated microscopy or flow cytometry systems have been used to screen for the presence of uropathogens.^11^, ^12^, ^13^, ^14^, ^15^, ^16^ Although the ability of flow cytometry to discriminate bacterial morphology has been reported, it has not been reported at present for automated microscopy systems.^11^, ^15^, ^16^ In this study, to contribute to the appropriate selection of antimicrobial therapy in the treatment of UTI, we evaluated the ability of the Atellica UAS800 automated microscopy system to detect bacteria and discriminate bacterial morphology and devised a predictive formula that can predict causative uropathogens of UTI using multiple logistic regression to improve its ability.

In the discrimination of bacterial morphology and vaginal contamination, the Atellica UAS800 showed only moderate performance with a single item, but the regression prediction formulas, which combined multiple items, showed high discrimination performance and excellent performance. Prediction formulas for the discrimination of bacterial morphology include BACr and EPI, which showed a negative regression coefficient, and age, which showed a positive regression coefficient. In other words, BACr and EPI have a low probability of identifying cocci when their values are high, but if patient age is high, the probability of identifying cocci increases. However, bacterial populations whose bacterial morphology is bacilli will contain a large number of bacteria such as *Lactobacillus* sp. that result from vaginal contamination. Therefore, as in the workflow proposed in this research, it will be necessary to combine each prediction formula to distinguish between bacterial morphology and vaginal contamination. Prediction formulas for the discrimination of vaginal contamination include EPI, which showed a positive regression coefficient, and MUC, which showed a negative regression coefficient. In other words, EPI would indicate a high probability of cocci when its value is high. However, the reason for MUC being included in the prediction formula is difficult to interpret.

In a study by Kim et al.^16^ using a UF‐5000 Flow Cytometric Analyzer, the sensitivity, specificity, PPV, and negative predictive value (NPV) were 91.7%, 90.0%, 95.4%, and 82.8%, respectively, in the Gram‐negative bacteria group when monobacterial samples containing ≥105 CFU/ml were used. However, the sensitivity, specificity, PPV, and NPV of the Gram‐positive bacteria group were 81.3%, 80.0%, 64.4%, and 90.6%, respectively, and their performance was inferior to that of the Gram‐negative bacteria. Besides, morphological instruments such as the iQ200 urine analyzer and cobas u 701 cannot differentiate bacilli and coccobacilli because they do not have items or flags to differentiate them.

The uropathogen prediction equation developed in this study using the Atellica UAS800 could not differentiate between the Cocci group and polymicrobial group, but it could differentiate between the Cocci or polymicrobial group and Bacilli group with 87.9% sensitivity and 85.4% specificity. This performance was also confirmed with the validation set. The PPV of bacilli and that of cocci or polymicrobials were 82% and 80%, respectively, which were comparable to those of flow cytometric analysis. In addition, because the Atellica UAS800 is a microscopy analyzer, it can detect squamous epithelial cells and thus is superior in differentiating vaginal contamination. These are very useful advantages of the Atellica UAS800.

This study has two limitations. First, we used fresh urine of outpatients suspected of having a UTI as the targeted material for this study, but we did not consider patient backgrounds. Therefore, it is possible that patients such as catheterized patients and pregnant women may have asymptomatic bacteriuria. However, as we usually do not consider the patient's background in routine urinalysis, the probability prediction equation of this study, which does not consider patient background, is optimal when used in daily workflow. Second, we analyzed only the data obtained from an automated microscopy system, but as the dip‐stick test is also performed in the actual inspection workflow, further improvement of the discrimination capacity is estimated by combining the results of both. In our previous study, the nitrite reaction of the dip‐stick test is a very useful item to distinguish between bacilli and cocci y.^17^

In conclusion, the Atellica UAS800 showed high performance in discriminating uropathogens using individual cell counts of bacilli and squamous epithelial cells, and its performance was further enhanced by combining the cell types. In addition, the probability prediction formula devised in this study could accurately discriminate the morphology of uropathogens of UTI and vaginal contamination. Incorporating this system into the general urinalysis system may contribute to more appropriate empiric therapy of UTI. Moreover, there is a likelihood that reducing the use of broad‐spectrum antimicrobial agents including fluoroquinolone antibacterials may inhibit the further emergence of drug‐resistant bacteria.

## CONFLICT OF INTEREST

None.

## AUTHOR CONTRIBUTION

All authors meet the ICMJE authorship criteria. AN, MK, and HY developed the trial design and contributed to the writing of the final manuscript. AN, TS, and NN were involved in data analysis and data interpretation.

## Data Availability

The data that support the findings of this study are available from the corresponding author upon reasonable request.
